# Correlation Analysis Among Genotype Resistance, Phenotype Resistance, and Eradication Effect After Resistance-Guided Quadruple Therapies in Refractory *Helicobacter pylori* Infections

**DOI:** 10.3389/fmicb.2022.861626

**Published:** 2022-03-07

**Authors:** Zijun Guo, Shuxin Tian, Weijun Wang, Yanbin Zhang, Jing Li, Rong Lin

**Affiliations:** ^1^Department of Gastroenterology, Union Hospital, Tongji Medical College, Huazhong University of Science and Technology, Wuhan, China; ^2^Department of Gastroenterology, The First Affiliated Hospital, Shihezi University, Shihezi, China

**Keywords:** *Helicobacter pylori*, antibiotic resistance, mutations, whole-genome sequencing, eradication

## Abstract

**Objectives:**

The antimicrobial resistance of *Helicobacter pylori* (*H. pylori*) in most countries and regions has increased significantly. It has not been fully confirmed whether the detection of *H. pylori* resistance gene mutation can replace antibiotic drug sensitivity test to guide the clinical personalized treatment. The objective of this study was to assess and compare the efficacy of different antimicrobial resistance-guided quadruple therapies in refractory *H. pylori*-infected individuals who had undergone unsuccessful prior eradication treatments.

**Methods:**

From January 2019 to February 2020, genotypic and phenotypic resistances were determined by polymerase chain reaction (PCR), whole genome sequencing (WGS) and broth microdilution test, respectively, in 39 *H. pylori*-infected patients who have failed eradication for at least twice. The patients were retreated with bismuth quadruple therapy for 14 days according to individual antibiotic resistance results. Eradication status was determined by the ^13^C-urea breath test.

**Results:**

The overall eradication rate was 79.5% (31/39, 95% CI 64.2–89.5%) in the intention-to-treat (ITT) analysis and 88.6% (31/35, 95% CI 73.5–96.1%) in the per- protocol analysis (PP) analysis. The presence of amoxicillin resistance (OR, 15.60; 95% CI, 1.34–182.09; *p* = 0.028), female sex (OR, 12.50; 95% CI, 1.10–142.31; *p* = 0.042) and no less than 3 prior eradication treatments (OR, 20.25; 95% CI, 1.67–245.44; *p* = 0.018), but not the methods for guiding therapy (*p* > 0.05) were associated with treatment failure. Resistance-guided therapy achieved eradication rates of more than 80% in these patients. The eradication rate of *H. pylori* in the phenotypic resistance-guided group was correlated well with genotype resistance-guided groups, including PCR and WGS.

**Conclusion:**

Culture or molecular method guiding therapy can enable personalized, promise salvage treatments, and achieve comparably high eradication rates in patients with refractory *H. pylori* infection. The detection of *H. pylori* resistance mutations has a good clinical application prospect.

**Protocol Study Register:**

[clinicaltrials.gov], identifier [ChiCTR1800020009].

## Introduction

*Helicobacter pylori* (*H. pylori*) is a Gram-negative microaerophilic bacterium that persistently colonizes the stomach of approximately 50% of the world’s population, equivalent to approximately 4.4 billion people (O’Connor et al., 2017). This infection establishes lifelong chronic progressive gastric inflammation, leading to a stepwise progression through gastric atrophy, intestinal metaplasia, and dysplasia, to the development of carcinoma (McColl, 2010). In the absence of an effective vaccine, treatment of chronic *H. pylori* infection has emerged as the main strategy for preventing subsequent gastric cancer development (Ford et al., 2014; Choi et al., 2018; Doorakkers et al., 2018).

Bismuth quadruple therapy, containing two antibiotics, plus bismuth and proton pump inhibitor (PPI) for 14 days, is recommended as a first-line empirical treatment for *H. pylori* infections by the Masstricht V Consensus (Malfertheiner et al., 2017). However, curing *H. pylori* infection has been proved remarkably difficult as the cure rates of empirical treatments are often <70% due to the increasing antimicrobial resistance (Malfertheiner et al., 2012; López-Góngora et al., 2015). And personalized treatment could be the main direction in the future, as it can significantly enhance the eradication rate, diminish the abuse of antibiotics, and avoid secondary antibiotic resistance (Fallone et al., 2016; Chey et al., 2017). Consequently, evaluating the resistance of *H. pylori* to drugs is of major clinical importance for guiding decisions about appropriate therapies in individuals and treatment policies in populations.

Susceptibility of *H. pylori* to antibiotics can be assessed by culture-based or molecular-based drug susceptibility testing (DST) techniques. Culture-based techniques are the standard DST for *H. pylori* and provide *in vitro* phenotypic susceptibly information (Miura and Hokari, 2012). However, this method is hampered by a relatively high rate of false negative, ranging from 45 to 90% (Mégraud and Lehours, 2007). Growth of *H. pylori* can be affected by many environmental factors and determination of the minimum inhibitory concentration (MIC) for *H. pylori* is cumbersome with a long turn-around-time, which limits their clinical application (Kjøller et al., 1991; Giorgio et al., 2016). Molecular-based methods rely mainly on the detection of specific *H. pylori* mutations encoding resistance. Globally, polymerase chain reaction (PCR)-based techniques have been developed to shorten the turn-around time and provide rapid detection of genotypic resistance in *H. pylori* (Cattoir et al., 2007). Besides, next-generation sequencing (NGS) refers to technologies that enable massively parallel sequencing (of DNA or RNA) to provide high-throughput genetic data at relatively low cost. Consequently, NGS would be applied in combination with culture for whole-genome sequencing (WGS), which is a powerful tool for antibiotic resistance prediction in *H. pylori* (Nezami et al., 2019).

However, in view of the correlation between genotypic resistance and phenotypic resistance and clinical treatment effect have not yet been fully elucidated, especially in China. Hence, we tried to further verify their consistency and the clinical application prospect of molecular techniques (PCR and WGS) guided eradication therapy. We conducted this trial by testing the resistance of antibiotics *in vitro*, detecting the mutation in the antibiotic resistance site by PCR and WGS, following up the efficacy of resistance-guided clinical eradication therapy, and analyzing the general data (demography, clinical diagnosis, and medical history, etc.) of the patients with refractory *H. pylori* infection.

## Materials and Methods

### Study Design and Population

This prospective, open-label trial was conducted in Wuhan Union Hospital and Pathogen Microbiology Laboratory, Tongji Medical College, Huazhong University of Science and Technology (clinicaltrials.gov identifier: ChiCTR1800020009). Between January 2019 and February 2020, a total of 39 *H. pylori*-infected patients who failed at least twice were prospectively enrolled in this study. The study protocol was approved by the Institutional Review Board of Tongji Medical College, Huazhong University of Science and Technology. Written informed consent was obtained from all patients prior to enrollment. We searched published works from PubMed, MEDLINE and the Cochrane Library for the terms “*H. pylori*,” “quadruple therapy,” “third-line,” “phenotypic resistance,” “genotypic resistance,” “polymerase chain reaction,” and “next generation sequencing” with no language or date limits. No publication of clinical trials that assessed and compared the efficacy of phenotypic resistance-guided and genotypic resistance-guided quadruple therapy in the at least third-line treatment of *H. pylori* infection was identified.

### Eligibility Criteria and Randomization

Patients were excluded from the study if any of the following criteria was present: (i) children and teenagers aged <18 years old; (ii) history of gastrectomy or non-curable malignancy; (iii) contraindication or previous allergic reaction to proton pump inhibitors (PPI), bismuth, or antibiotics (clarithromycin, amoxicillin, metronidazole, levofloxacin, furazolidone, and tetracycline); (iv) pregnant or lactating women or severe concurrent diseases; (v) any of the three methods for detecting antibiotic resistance failed. Before *H. pylori* eradication, patients were randomly assigned to three groups using a random-number chart: (i) treated according to culture-based antibiotic susceptibility testing; (ii) treated according to PCR-based testing; (iii) treated according to WGS.

### Determination of *Helicobacter pylori* Status

Before enrollment, the status of *H. pylori* infection was determined by the ^13^C-urea breath test (^13^C-UBT) or ^14^C-urea breath test (^14^C-UBT). Patients with either positive ^13^C-UBT or positive ^14^C-UBT at least twice were defined as refractory to previous treatment and were eligible for enrollment. Oesophago-gastro-duodenoscopy with biopsies from gastric antrum (two pieces for *H. pylori* culture, one pieces for PCR test and necessary number of pieces for histology) were performed for all patients. After treatment, *H. pylori* status was determined by ^13^C-UBT or ^14^C-UBT ≥4 weeks after the completion of eradication therapy. Successful eradication of *H. pylori* was defined as a negative ^13^C-UBT or ^14^C-UBT result. All patients were asked to stop proton pump inhibitors (PPI) for ≥2 weeks and antibiotics or bismuth for ≥4 weeks before endoscopy examination. Positive and negative results of ^13^C-UBT were defined according to the results of previous study as a cut-off value of ≥5 and <2.5‰, respectively (Chen et al., 2003). Patients with uncertain results received another ^13^C-UBT until the result was conclusive.

### Determination of Phenotypic and Genotypic Resistance

#### Phenotypic Resistance: Culture-Based Drug Susceptibility Testing

**FIGURE 1:**
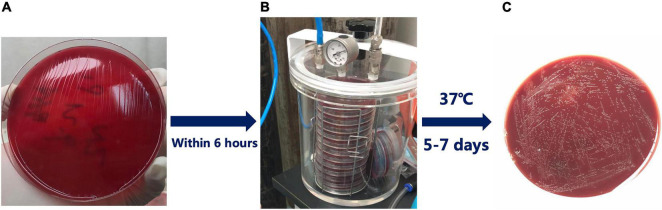
Isolation of *Helicobacter pylori* (*H. pylori*) from clinical biopsy specimens. **(A)** The biopsy specimens were applied to chocolate agar plates containing 10% sheep blood; **(B)** Sealing device for culturing *H. pylori*; **(C)** The transparent color colonies of *H. pylori* after successful culture.

The biopsy specimens were cultured on chocolate agar plates containing 10% sheep blood and incubated under microaerobic conditions (5% O_2_, 10% CO_2_, and 85% N_2_) for 5–7 days (Figure 1). Phenotypic resistance was determined by the broth microdilution test if strains were available. *H. pylori* was inoculated onto antibiotic-containing broth medium supplemented with 5% defibrinated sheep blood. *H. pylori* ATCC 26695 was used as the quality control strain. The MIC of each antibiotic was determined after 72 h of incubation. The breakpoints for amoxicillin, clarithromycin, levofloxacin, tetracycline, metronidazole, and furazolidone resistance were defined as ≥0.5, ≥1, ≥1, ≥1, ≥8, and ≥2 μg/mL, respectively (Midolo et al., 1997; Liou et al., 2010; Chung et al., 2017). Each experiment was performed in triplicate, and experiments were repeated at least three times per strain.

#### Genotypic Resistance: Polymerase Chain Reaction-Based Assays

The DNA of *H. pylori* was extracted from gastric biopsy tissues using DNA extraction kit (Gentra DNA Purification Kit, QIAGEN, Hilden, Germany) according to the manufacturer’s instructions. The gene fragment of *H. pylori* correlated with different antibiotics was amplified by PCR with specific primers and then sequenced (Table 1). As established by previous studies, the presence of the specific variants is listed (Supplementary Table 1). All PCR mixtures were prepared in a final volume of 25 μL containing 50 ng DNA from the samples served as the template for PCR performed in a thermal cycler (Master cycler gradient, Eppendorf, Germany), 10 μM of each primer, 8.5 μL ddH_2_O, 2 μL template DNA, 12.5 μL Mix of Taq DNA polymerase. Thermal amplification of PCR products was performed at 94°C for 4 min and then for 35 cycles of 94°C for 1 min, 55°C for 40 s, 72°C for 1 min, and a final extension at 72°C for 7 min, with a final hold at 10°C in a PCR thermal cycler (Master cycler gradient). The PCR amplified products (10 μL) were subjected to electrophoresis in 1.5% agarose gel in 1 × TBE buffer at 80 V for 30 min stained with a solution of ethidium bromide (EMD Millipore, Billerica, MA, United States). And examined under ultraviolet illumination (Cleaver Scientific Ltd., Rugby, United Kingdom). The genotypic information of *H. pylori* ATCC 26695 was used as reference.

**TABLE 1:** Polymerase chain reaction primers used for detecting mutations of *H. pylori* strains in this study.

Antibiotics	Genes	Primer sequence (5′–3′)
clarithromycin	23S rRNA	F: ACAGCACTTTGCCAACTCGTAA R: GCTTGTGCCATTACACTCAACTTG
amoxicillin	PBP-1A	F: GCCATTCTTATCGCTCAAGTTTGG R: ATCGCTAAAATGTTACGCATGAAATACG
levofloxacin	gyrA	F: TGGGGATTGATTCTTCTATTGAAGA R: TGCACTAAAGCGTCTATGATTTCA
Tetracycline	16S rRNA	F: TCCGTAGAGATCAAGAGGAATACTCATTG R: TCACCGCAACATGGCTGATTTG
furazolidone	oorD	F: GGCTTGCCTGGAAATCCTGTAG R: AACCGATTTGCTCCACTTTCAATGA
	porD	F: GCAAGAAGTCATTGACGC R: GGGGTGATAGGATAGGCT
metronidazole	rdxA	F: GCAGGAGCATCAGATAGT R: GGGTGATTTCTTGGTTGC

#### Genotypic Resistance: Library Preparation and Whole Genome Sequencing of *Helicobacter pylori* Strains

Library preparation was performed using the Qiagen^®^ QIAseq FX DNA Kit (Qiagen, Hilden, Germany) according to the manufacturer’s recommendations. Furthermore, next-generation sequencing (MiSeq next-generation sequencer; Illumina, San Diego, CA, United States) was used to analyze all strains for mutation status of 23S rRNA, PBP-1A, gyrA, 16S rRNA, oorD, and porD genotypes. The BLAST algorithm implemented in the CLC Genomics Workbench software (ver. 11; Qiagen NV, Venlo, Netherlands) was used for the analysis. The sequences of hp0425, hp0597, hp0701, hp0374, hp0954, oorD, and porD of the strain 26695 (GenBank accession number AE000511.1) were used as queries to obtain the 23S rRNA, PBP-1A, gyrA, 16S rRNA, rdxA, oorD, and porD sequences, respectively, from the next-generation sequencing data. Subsequently, each codon of the strains was compared to the reference sequence hp 26699 using our original PERL script and confirmed by visual inspection. Discrepancies found between the strains and reference sequence, which was included in the variants of Supplementary Table 1, was considered as variants related to antibiotic resistance.

### Susceptibility-Guided Intervention and Assessment of Adverse Effects

Treatment recommendations based on susceptibility testing were given on request in line with guideline from the fifth national consensus report on the treatment of *H. pylori* infection (Liu et al., 2018; Table 2). Six antibiotics are available for use in combined therapies in *H. pylori* eradication regimes: clarithromycin, amoxicillin, levofloxacin, metronidazole, tetracycline, and furazolidone. Patients were treated with quadruple therapy containing 30 mg of lansoprazole, 200 mg of pectin bismuth and 2 combined antibiotics for 14 days, according to the phenotypic or genotypic resistance determined using biopsy specimens or isolated strains. If the determined antibiotics for *H. pylori* sensitivity were less than 2 or not included in the regimens (Table 2), the patients would be treated empirically according to their previous medication history to avoid the reuse of unnecessary antibiotics. Patients were informed of the common adverse effects and were asked to record their symptoms during treatment.

**TABLE 2:** Recommended combined regimens of *H. pylori* in China.^†^

Regimen^‡^	Drug 1	Drug 2
1	AMX, 1000 mg, bid	CLR, 500 mg, bid
2	AMX, 1000 mg, bid	LVX, 500 mg, qd/200 mg, bid
3	AMX, 1000 mg, bid	FZD, 100 mg, bid
4	TET, 500 mg, tid/qid	MTZ,400 mg, tid/qid
5	TET, 500 mg, tid/qid	FZD, 100 mg, bid
6	AMX, 1000 mg, bid	MTZ,400 mg, tid/qid
7	AMX, 1000 mg, bid	TET, 500 mg, tid/qid

*CLR, clarithromycin; AMX, amoxicillin; LVX, levofloxacin; MTZ, metronidazole; TET, tetracycline; FZD, furazolidone. Qd, once daily; bid, twice daily; tid, three-times daily; qid, four-times daily.*

*^†^Table adapted from the fifth national consensus report on the treatment of Helicobacter pylori infection, Chin J. Gastroenterol., 2017, Vol. 22, No. 6.*

*^‡^Standard dose (PPI + bismuth) (bid, orally half an hour before a meal) + 2 antibacterial drugs (orally after a meal). The standard dose of PPI is lansoprazole 30 mg; the standard dose of bismuth is 200 mg of pectin bismuth.*

### Statistical Analysis

The statistical analyses were performed using the SPSS 26.0 statistical software for Windows. Categorical data were compared using the Chi-square test or Fisher’s exact test, as appropriate. Continuous data were compared with Student’s *t*-test and expressed as the mean (SD). The kappa coefficient was used to assess the agreement between genotypic resistance and phenotypic resistance. Logistic regression analysis was performed to analyze factors affecting the eradication rates. The statistical significance level was set at 0.05. And odds ratios (ORs) and 95% confidence intervals (CIs) were calculated.

## Results

### Baseline Characteristics of Patients

**FIGURE 2:**
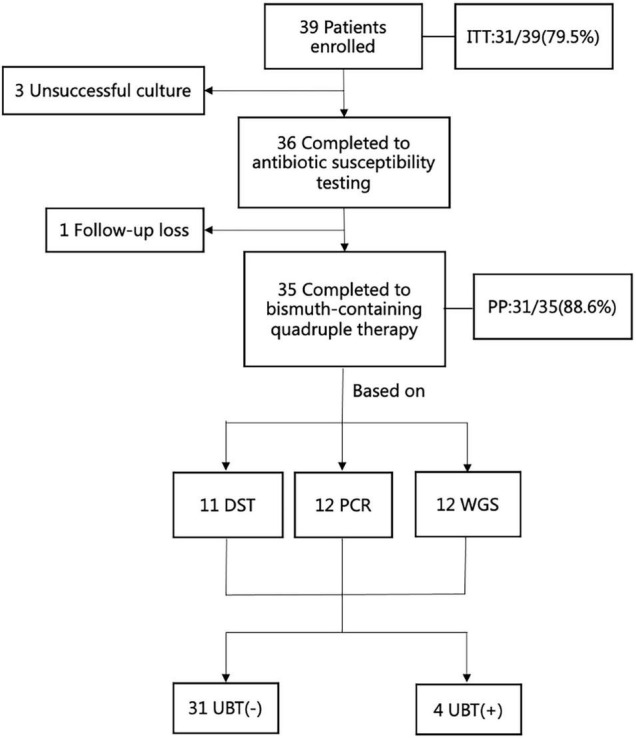
The flow chart of enrollment in this study. ITT, intention -to-treat; PP, per-protocol; UBT, urea breath test; DST, drug susceptibility testing; PCR, polymerase chain reaction; WGS, whole genome sequencing.

Thirty-six *H. pylori* strains were isolated from 39 patients, and eventually 35 patients were analyzed for *H. pylori* eradication as a result of 1 follow-up loss (Figure 2). The culture-based DST was successfully determined in all of them with broth microdilution testing. The genotypic resistance was successfully determined using PCR tests as well as WGS in the same thirty-six patients above. There were 17 male patients and 19 female patients. The average age of the patients was 47 years old, ranging from 27 to 62 years old. Of the 36 patients with adequate information regarding their medication history, 100% (36/36) received clarithromycin and amoxicillin in their previous eradication regimens, compared with 11.1% (4/36), 8.3% (3/36), 2.8% (1/36), and 16.7% (6/36) for levofloxacin, metronidazole, tetracycline, and furazolidone, respectively. The baseline characteristics of patients included in the study are summarized (Table 3). Among three groups, all of baseline characteristics are of no significance.

**TABLE 3:** Baseline characteristics of patients included in the study (*n* = 36).

Characteristic	Basis for treatment			*P* value*
	DST (*n* = 12), *n* (%)	PCR (*n* = 12), *n* (%)	WGS (*n* = 12), *n* (%)	
Age (years), mean (range)	42 (27–62)	43 (26–60)	45 (29–63)	0.137
Gender, male	5 (41.67)	7 (58.33)	5 (41.67)	0.640
Current smoker	3 (25.00)	5 (41.67)	2 (16.67)	0.381
Endoscopic findings				0.449
peptic ulcer	2 (16.67)	3 (25.00)	0 (0.00)	
intestinal metaplasia	4 (33.33)	3 (25.00)	4 (33.33)	
atrophic gastritis	3 (25.00)	4 (33.33)	6 (50.00)	
others	3 (25.00)	2 (16.67)	2 (16.67)	
Number of prior eradication treatments				0.074
2	10 (83.33)	9 (75.00)	5 (41.67)	
≥3	2 (16.67)	3 (25.00)	7 (58.33)	
Prior treatment				
clarithromycin	12 (100.0)	12 (100.0)	12 (100.0)	NA
amoxicillin	12 (100.0)	12 (100.0)	12 (100.0)	NA
levofloxacin	1 (8.33)	2 (16.67)	1 (8.33)	0.766
metronidazole	1 (8.33)	0 (0.00)	2 (16.67)	0.336
tetracycline	1 (8.33)	0 (0.00)	0 (0.00)	0.324
furazolidone	1 (8.33)	4 (33.33)	1 (8.33)	0.183
Virulence factors				
cagA positive	12 (100.0)	11 (91.67)	11 (91.67)	0.432
ureA/ureB positive	12 (100.0)	12 (100.0)	12 (100.0)	NA

*DST, drug susceptibility testing; PCR, polymerase chain reaction; WGS, whole genome sequencing; cagA, cytotoxin-associated gene A; vacA, gene encoding vacuolating cytotoxin A.*

*All P values were for testing the difference between DST, PCR, and WGS subgroups and were calculated by Student’s t test or Fisher’s exact test.

### Prevalence of Antibiotic Resistance

As all patients had undergone prior unsuccessful eradication treatments, the proportions of *H. pylori* antimicrobial resistant to clarithromycin, levofloxacin and metronidazole were markedly high and associated with the number of treatment failures (Table 4). However, none of the strains tested was resistant to furazolidone or tetracycline. And the prevalence of resistance to clarithromycin, levofloxacin, amoxicillin, and metronidazole was 91.7% (33/36), 50.0% (18/36), 25.8 (8/36), 41.7 (15/36), respectively. Markedly, more double resistant *H. pylori* isolates were observed in these patients (91.7%) (Table 4).

**TABLE 4:** Antimicrobial resistance of *H. pylori* isolated from 36 patients with unsuccessful prior eradication treatments.

Resistant to	Number of prior eradication treatments				Overall (*n* = 36)	
	2 (*n* = 24)		≥3 (*n* = 12)			
	*n*	%	*n*	%	*n*	%
CLR*	21	87.5	12	100.0	33	91.7
LVX**	11	45.8	7	58.3	18	50.0
MTZ***	9	37.5	6	50.0	15	41.7
AMX**	5	20.8	3	25.0	8	25.8
CLR/LVX^†^	10	41.7	6	50.0	16	44.4
CLR/MTZ^†^	7	29.2	4	33.3	11	30.6
CLR/AMX^†^	1	4.2	2	16.7	3	8.3
AMX/MTZ^†^	2	8.3	1	8.3	3	8.3
CLR/LVX/AMX^††^	0	0.0	1	100.0	1	2.8
CLR/LVX/MTZ/AMX^†††^	0	0.0	1	100.0	1	2.8

*CLR, clarithromycin; AMX, amoxicillin; LVX, levofloxacin; MTZ, metronidazole.*

*Resistance included monoresistance, double resistance, triple resistance, and quadruple resistance.

**Resistance included double resistance, triple resistance, and quadruple resistance.

***Resistance included double resistance and quadruple resistance.

*^†^Double resistance.*

*^††^Triple resistance.*

*^†††^Quadruple resistance.*

The mutant genes of *H. pylori* isolated from 36 patients with unsuccessful prior eradication treatments according to PCR or WGS were shown (Table 5). The proportions of mutant 23S rRNA, PBP-1A, gyrA, and rdxA using PCR were 88.9% (32/36), 22.2% (8/36), 47.2% (17/36), and 44.4% (16/36), respectively. While the proportions of mutant 23S rRNA, PBP-1A, gyrA, and rdxA using whole genome sequencing were 81.7% (33/36), 22.2% (8/36), 50.0% (18/36), and 50.0% (18/36), respectively. None of mutations were found either in 16S rRNA gene or oorD/porD gene (Table 5).

**TABLE 5:** Mutant genes of *H. pylori* isolated from 36 patients with unsuccessful prior eradication treatments.

Antibiotic	Mutant genes	Genotypic resistance testing	

		**PCR, *n* (%)**	**WGS, *n* (%)**
Clarithromycin	23S rRNA	32 (88.9)	33 (91.7)
Amoxicillin	PBP-1A	8 (22.2)	8 (22.2)
Levofloxacin	gyrA	17 (47.2)	18 (50.0)
Metronidazole	rdxA	16 (44.4)	18 (50.0)
Tetracycline	16S rRNA	0 (NA)	0 (NA)
Furazolidone	oorD/porD	0 (NA)	0 (NA)

*PCR, polymerase chain reaction; WGS, whole genome sequencing.*

The agreement between culture-based DST results and identified point mutations by PCR or WGS was shown (Table 6), conferring resistance to CLR, LVX, AMX, and MTZ. The genotypic resistance determined using PCR in biopsy specimens correlated well with the phenotypic DST determined using strains for both clarithromycin and levofloxacin, amoxicillin, as well as metronidazole (*k* > 0.80) (Table 6). Overall, there was a comparatively high congruence of >90% between phenotypic DST results for clarithromycin, levofloxacin, amoxicillin, and metronidazole and SNPs identified by WGS in the 23S rRNA, gyrA, PBP-1A, and rdxA genes.

**TABLE 6:** Correlations between phenotypic resistance by DST and genotypic resistance determined by PCR and WGS.

Phenotypic	Genotypic							
	23S rRNA (PCR)		*P* *	*k* coefficient	23S rRNA (WGS)		*P* *	*k* coefficient
CLR MIC	W	M			W	M		
S	3	0	<0.001	0.842	3	0	<0.001	1.000
R	1	32			0	33		
	
**LVX MIC**	**gyrA** **(PCR)**		*P* *	***k* coefficient**	**gyrA** **(WGS)**		*P* *	***k* coefficient**
	**W**	**M**			**W**	**M**		

S	17	1	<0.001	0.833	18	0	<0.001	0.944
R	2	16			1	17		
	
**AMX MIC**	**PBP-1A** **(PCR)**		*P* *	***k* coefficient**	**PBP-1A** **(WGS)**		*P* *	***k* coefficient**
	**S**	**R**			**W**	**M**		

S	27	1	<0.001	0.839	28	0	<0.001	0.916
R	1	7			1	7		
	
**MTZ MIC**	**rdxA** **(PCR)**		*P* *	***k* coefficient**	**rdxA** **(WGS)**		*P* *	***k* coefficient**
	**S**	**R**			**S**	**R**		

S	20	1	<0.001	0.943	18	3	<0.001	0.833
R	0	15			0	15		

*DST, drug susceptibility testing; PCR, polymerase chain reaction; WGS, whole genome sequencing; MIC, minimum inhibitory concentration; CLR, clarithromycin; AMX, amoxicillin; LVX, levofloxacin; MTZ, metronidazole; S, susceptible; R, resistant; W, wild type; M, mutant.*

*All P values were calculated by Fisher’s exact test.

### Eradication Rates and Factors Affecting the Efficacy

In the present study, four antibiotic combination regimens were applied in the quadruple therapy, TET + FZD, TET + MTZ, AMX + FZD, and AMX + LVX, respectively. Moreover, AMX + FZD was the main regimen used in the quadruple therapy for patients in the DST and WGS groups, while AMX + LVX was the main regimen used in the quadruple therapy for patients in the PCR group (Table 7). The overall eradication rate was 79.5% (31/39, 95% CI 64.2–89.5%) in the ITT analysis and 88.6% (31/35, 95% CI 73.5–96.1%) in the PP analysis. Factors possibly related to eradication failure are summarized (Table 8). Furthermore, the four patients with failed eradication consisted of one from DST group, two from PCR group and one from WGS group with treatment regimens of TET + FZD, TET + MTZ, AMX + LVX, and TET + MTZ, respectively. In univariate analysis, there were statistically significant clinical factors associated with eradication failure, including male: female ratio (*p* = 0.042), number of prior eradication treatments (*p* = 0.018) and AMX resistance (*p* = 0.028). Besides, we also found no statistical difference in eradication failure rates related to age, cigarette smoking, alcohol intake, diabetes, hypertension, high salt diet, family history of GC, method for guiding therapy, endoscopic findings, CLR resistance, LVX resistance, MTZ resistance, CagA, and VacA (*p* > 0.05). It is note-worthy that the eradication rate based on DST, PCR and WGS was 90.9% (10/11), 83.3% (10/12), and 91.7% (11/12), respectively, which indicated that genotypic resistance-guided therapy might achieve satisfactory results as same as phenotypic resistance-guided therapy. Furthermore, logistic regression analysis revealed that female sex (OR, 12.50; 95% CI, 1.10–142.31; *p* = 0.042), not less than 3 prior eradication treatments (OR, 20.25; 95% CI, 1.67–245.44; *p* = 0.018) and AMX resistance (OR, 15.60; 95% CI, 1.34–182.09; *p* = 0.028) were significantly associated with eradication failure (Table 9).

**TABLE 7:** Antibiotic combination regimens in three groups of patients in the quadruple therapy.

Regimen	Basis for treatment			Overall (*n* = 36), *n* (%)

	**DST (*n* = 12), *n* (%)**	**PCR (*n* = 12), *n* (%)**	**WGS (*n* = 12), *n* (%)**	
TET + FZD	1 (8.3)	2 (16.7)	1 (8.3)	4 (11.1)
TET + MTZ	1 (8.3)	1 (8.3)	2 (16.7)	4 (11.1)
AMX + FZD	7 (58.3)	3 (25.0)	6 (50.0)	16 (44.4)
AMX + LVX	3 (25.0)	6 (50.0)	3 (25.0)	12 (33.3)

*AMX, amoxicillin; LVX, levofloxacin; MTZ, metronidazole; TET, tetracycline; FZD, furazolidone. DST, drug susceptibility testing; PCR, polymerase chain reaction; WGS, whole genome sequencing.*

**TABLE 8:** Clinical characteristics in eradication success and failure groups (*n* = 35).

Factor	Success group (*n* = 31), *n* (%)	Failure group (*n* = 4), *n* (%)	*P* value*
Age, yr.	42.6 ± 11.0	42.5 ± 9.7	0.993
Male: Female ratio	25:6 (80.6:19.4)	1:3 (25.0:75.0)	0.044
Cigarette smoking	9 (29.0)	1 (25.0)	0.681
Alcohol intake	19 (38.7)	1 (25.0)	0.522
Diabetes	3 (9.7)	0 (0.0)	0.687
Hypertension	8 (25.8)	1 (25.0)	0.732
High salt diet	19 (61.3)	2 (50.0)	0.530
Family history of GC	2 (6.5)	0 (0.0)	0.782
Method for guiding therapy			0.788
DST	10 (32.3)	1 (25.0)	
PCR	10 (32.3)	2 (50.0)	
WGS	11 (31.4)	1 (25.0)	
Number of prior eradication treatments			0.019
2	27 (87.1)	1 (25.0)	
≥3	4 (12.9)	3 (75.0)	
Endoscopic findings			0.478
Non-atrophic	11 (35.5)	2 (50.0)	
Atrophic	20 (64.5)	2 (50.0)	
CLR resistance^†^	29 (93.5)	3 (75.0)	0.313
AMX resistance^†^	5 (16.1)	3 (75.0)	0.030
LVX resistance^†^	10 (55.6)	2 (50.0)	0.632
MTZ resistance^†^	9 (60.0)	3 (75.0)	0.525
CagA			0.218
Negative	1 (3.2)	1 (25.0)	
Positive	30 (96.8)	3 (75.0)	
VacA			0.732
m1	8 (25.8)	1 (25.0)	
m2/m1 + m2	23 (74.2)	3 (75.0)	

*GC, gastric cancer; DST, drug susceptibility testing; PCR, Polymerase chain reaction; WGS, whole genome sequencing; CLR, clarithromycin; AMX, amoxicillin; LVX, levofloxacin; MTZ, metronidazole; cagA, cytotoxin-associated gene A; vacA, gene encoding vacuolating cytotoxin A.*

*All P values were for testing the difference between Success group and failure group and were calculated by Student’s t test or Fisher’s exact test.

*^†^The resistance determined by antibiotic susceptibility testing.*

**TABLE 9:** Logistic regression analysis for *Helicobacter pylori* eradication failure.

Variable	Unadjusted OR	95% CI	*P* value*
Female sex	12.50	1.10–142.31	0.042
Not less than 3 prior eradication treatments	20.25	1.67–245.44	0.018
AMX resistance^†^	15.60	1.34–182.09	0.028

*OR, odds ratio; CI, confidence interval; AMX, amoxicillin.*

*All P values were calculated by logistic regression analysis.

*^†^The resistance determined by antibiotic susceptibility testing.*

### The Side Effects of *Helicobacter pylori* Eradication

Of the 35 participants, 15 participants (42.9%) complained of adverse events after 14 days of quadruple therapy. The side effects were not significantly different between the success group and failure group (Supplementary Table 2). In addition, the combination of TET and FZD was significantly associated with side effects of *H. pylori* eradication (*p* = 0.026), but the presence of TET and FZD alone was not associated with the development of adverse reactions (TET + MTZ, *p* = 0.581; AMX + FZD, *p* = 0.557) (Supplementary Table 3).

## Discussion

The work presented above lead to several novel findings relevant to the optimization of refractory *H. pylori* eradication. This was the first study to compare the prevalence of refractory antibiotic resistance of six antibiotics in *H. pylori* by using phenotypic DST and PCR-based assays as well as WGS-based assays at the same time. We further showed that resistance-guided modified quadruple therapy was effective (>80%) in the treatment of refractory *H. pylori* infection. More importantly, the eradication rates appeared to be same in patients treated with genotypic resistance-guided therapy as compared with those treated with phenotypic resistance-guided therapy. Besides, we found that not less than 3 prior eradication treatments, the presence of amoxicillin resistance and female sex were associated with treatment failure in patients treated with resistance-guided quadruple therapy.

In the present study, we have demonstrated that the genotypic resistance determined using gastric biopsy specimens as well as WGS correlated well with the phenotypic resistance determined in *H. pylori* strains. We could show a clear correlation between the occurrence of mutations in the 23S rRNA, gyrA, PBP-1A, and rdxA genes of *H. pylori* and clarithromycin, fluoroquinolone, amoxicillin, and metronidazole resistance, respectively. In a prospective study, Konrad also found that clarithromycin and levofloxacin gene resistance is consistent with the phenotypic resistance of *H. pylori*, which was consistent with our results (Egli et al., 2020). Few molecular diagnostic tests were generally performed for detecting the tetracycline or furazolidone resistance on gastric tissue samples because this may be of a little significance for initial antibiotic selection at the beginning of therapy, owing to the well documented low resistance rate to tetracycline/furazolidone (Fiorini et al., 2018; Palmitessa et al., 2020). And in this study, interestingly, neither phenotypic resistance nor genotypic resistance appeared in tetracycline and furazolidone, which demonstrated that it might be reasonable to use tetracycline and furazolidone empirically even in patients who have failed multiple treatment.

During this study, we realized that the efficacies appeared to be similar in patients treated with therapies guided by three types of testing as *p* value was of no significance among them. The successful cultivation from gastric biopsy specimens and DST are the gold standards for the phenotypic sensitivity test of *H. pylori*, which provide reliable information for the development of personalized clinical programs (Gerrits et al., 2006). However, this method is challenging due to the pathogen’s fastidious growth requirements for laboratory environment, taking up to at least 10 days to obtain results (Gerrits et al., 2006). Besides, culture-based methods could be hampered by a few technical factors, such as quality of the clinical specimen, occurrence of microbial commensal flora and inappropriate transport conditions (Cuadrado-Lavín et al., 2012). And most clinical laboratories, especially small and medium-sized hospitals in China, could not carry out this clinical project on a large scale, and most patients are not willing to wait for 2 weeks to be treated. Therefore, more rapid, and convenient molecular detection technology is ready. The determination of genotypic resistance using biopsy specimens is more expedient (culture not needed) and speedier, and it is easier to transfer the specimen (Cui et al., 2021) and has a higher success rate compared with traditional susceptibility tests (100.0 versus 92.3% in the present study).

Moreover, WGS delivers a more complete picture of resistance determinants present in a clinical isolate than PCR that can only examine a limited number of nucleotide positions (Binh et al., 2015). And the relevance of new polymorphisms can easily be assessed by later retrospective analysis of WGS data (Binh et al., 2014). However, WGS is obviously costlier than qPCRs. Anyway, genotypic resistance-guided quadruple therapies might be practical strategies in the treatment of refractory *H. pylori* infection in future clinical practice.

We demonstrated that female sex, not less than 3 prior eradication treatments and the presence of AMX resistance were associated with treatment failure in patients treated with resistance-guided quadruple therapy. However, the use of AMX under resistance guidelines remains acceptable in the third-line treatment of AMX-sensitive patients, as the rate of resistance to AMX remained relatively low in patients who have failed at least two eradication therapies. Besides, patients with AMX resistant can use a combination regimen containing TET with a lower resistance rate under resistance guidance. Recommended antimicrobial combinations for bismuth quadruple regimens include: (i) TET + MTZ; (ii) TET + FZD; (iii) TET + LVX. In addition, Vonoprazan, a new potassium competitive acid blocker, can be used to replace PPI. Vonoprazan is stable, highly efficacious, and less affected by CYP2C19 gene polymorphism, which is beneficial to improve *H. pylori* eradication rate (Akazawa et al., 2016). There are several reports that female gender can influence *H. pylori* eradication (Binh et al., 2014, 2015). One study suggested that there might be a difference in gastric physiology between males and females (Akazawa et al., 2016). Our study showed that the female gender is an unfavorable factor affecting eradication. However, the cause of gender differences in the eradication rate needs further research. Besides, data has shown that after just one unsuccessful therapy, resistance to clarithromycin rose to 60%, and further vain treatment attempts resulted in resistance as high as 80% (Wüppenhorst et al., 2014; Yahaghi et al., 2014; Thung et al., 2016). Although we found the efficacy of eradication was affected by these three factors, the results should be validated in further studies because of the wide CIs, which indicated small case numbers for these three variables.

However, this study had some limitations. First, it was a single-arm prospective research which did not include the relatively large sample size in third-line therapy. Second, the prevalence of CLR and AMX resistance is higher than average status because of nearly all of patients enrolled had prior treatments in Wuhan Union Hospital, where the empirically first-line regimen included antibiotic combination of CLR and AMX. Third, we should focus on predicating new point mutations of drug resistance in *H. pylori* based on the bacterium’s genome using next generation sequencing (NGS) technology in the future.

In conclusion, the results from this study show that genotypic resistance-guided quadruple therapy can achieve comparably high eradication rates as compared with phenotypic resistance-guided therapy in the treatment of refractory *H. pylori* infection. The detection of *H. pylori* resistance genes could be a good clinical application in the eradication of *H. pylori*.

## Data Availability Statement

The datasets presented in this study can be found in online repositories. The names of the repository/repositories and accession number(s) can be found in the article/Supplementary Material.

## Ethics Statement

The studies involving human participants were reviewed and approved by the Institutional Review Board of Tongji Medical College, Huazhong University of Science and Technology. The patients/participants provided their written informed consent to participate in this study.

## Author Contributions

RL and ZG designed the project. ST and WW supplied samples. ZG performed all experiments, collected, and analyzed data. YZ and JL followed the participants. ZG, ST, and WW drafted and revised the manuscript. All authors read and approved the final manuscript.

## Conflict of Interest

The authors declare that the research was conducted in the absence of any commercial or financial relationships that could be construed as a potential conflict of interest.

## Publisher’s Note

All claims expressed in this article are solely those of the authors and do not necessarily represent those of their affiliated organizations, or those of the publisher, the editors and the reviewers. Any product that may be evaluated in this article, or claim that may be made by its manufacturer, is not guaranteed or endorsed by the publisher.

## Abbreviations

*H. pylori*: *Helicobacter pylori*
PPI: proton pump inhibitor
DST: drug susceptibility testing
MIC: minimum inhibitory concentration
PCR: polymerase chain reaction
NGS: next-generation sequencing
WGS: whole-genome sequencing
^13^C-UBT: ^13^C-urea breath test
^14^C-UBT: ^14^C-urea breath test
ORs: odds ratios
CIs: confidence intervals
CLR: clarithromycin
AMX: amoxicillin
LVX: levofloxacin
TET: tetracycline
FZD: furazolidone.

## References

[B1] Akazawa Y. Fukuda D. Fukuda Y. (2016). Vonoprazan-based therapy for *Helicobacter pylori* eradication: experience and clinical evidence. *Therap Adv. Gastroenterol.* 9 845–852. 10.1177/1756283X16668093 PMC5076777 27803739

[B2] Binh T. T. Shiota S. Suzuki R. Matsuda M. Trang T. T. Kwon D. H. (2014). Discovery of novel mutations for clarithromycin resistance in *Helicobacter pylori* by using next-generation sequencing. *J. Antimicrob Chemother.* 69 1796–1803. 10.1093/jac/dku050 24648504 PMC4054984

[B3] Binh T. T. Suzuki R. Trang T. T. Kwon D. H. Yamaoka Y. (2015). Search for novel candidate mutations for metronidazole resistance in *Helicobacter pylori* using next-generation sequencing. *Antimicrob Agents Chemother.* 59 2343–2348. 10.1128/AAC.04852-14 25645832 PMC4356779

[B4] Cattoir V. Nectoux J. Lascols C. Deforges L. Delchier J. C. Megraud F. (2007). Update on fluoroquinolone resistance in *Helicobacter pylori*: new mutations leading to resistance and first description of a gyrA polymorphism associated with hypersusceptibility. *Int. J. Antimicrob Agents* 29 389–396. 10.1016/j.ijantimicag.2006.11.007 17303392

[B5] Chen T. S. Chang F. Y. Chen P. C. Huang T. W. Ou J. T. Tsai M. H. (2003). Simplified 13C-urea breath test with a new infrared spectrometer for diagnosis of *Helicobacter pylori* infection. *J. Gastroenterol. Hepatol.* 18 1237–1243. 10.1046/j.1440-1746.2003.03139.x 14535979

[B6] Chey W. D. Leontiadis G. I. Howden C. W. Moss S. F. (2017). ACG clinical guideline: treatment of *Helicobacter pylori* infection. *Am. J. Gastroenterol.* 112 212–239. 10.1038/ajg.2016.563 28071659

[B7] Choi I. J. Kook M. C. Kim Y. I. Cho S. J. Lee J. Y. Kim C. G. (2018). *Helicobacter pylori* therapy for the prevention of metachronous gastric Cancer. *N. Engl. J. Med.* 378 1085–1095. 10.1056/NEJMoa1708423 29562147

[B8] Chung J. W. Kim S. Y. Park H. J. Chung C. S. Lee H. W. Lee S. M. (2017). *In vitro* activity of diphenyleneiodonium toward multidrug-resistant *Helicobacter pylori* strains. *Gut Liver* 11 648–654. 28750485 10.5009/gnl16503 PMC5593327

[B9] Cuadrado-Lavín A. Salcines-Caviedes J. R. Carrascosa M. F. Mellado P. Monteagudo I. Llorca J. (2012). Antimicrobial susceptibility of *Helicobacter pylori* to six antibiotics currently used in Spain. *J. Antimicrob Chemother.* 67 170–173. 10.1093/jac/dkr410 21965436

[B10] Cui R. Song Z. Suo B. Tian X. Xue Y. Meng L. (2021). Correlation analysis among genotype resistance, phenotype resistance and eradication effect of *Helicobacter pylori*. *Infect Drug Resist.* 14 1747–1756. 10.2147/IDR.S305996 34012273 PMC8127322

[B11] Doorakkers E. Lagergren J. Engstrand L. Brusselaers N. (2018). *Helicobacter pylori* eradication treatment and the risk of gastric adenocarcinoma in a Western population. *Gut* 67 2092–2096. 29382776 10.1136/gutjnl-2017-315363

[B12] Egli K. Wagner K. Keller P. M. Risch L. Risch M. Bodmer T. (2020). Comparison of the diagnostic performance of qPCR, sanger sequencing, and whole-genome sequencing in determining clarithromycin and levofloxacin resistance in *Helicobacter pylori*. *Front. Cell Infect Microbiol.* 10:596371. 10.3389/fcimb.2020.596371 33392106 PMC7773895

[B13] Fallone C. A. Chiba N. van Zanten S. V. Fischbach L. Gisbert J. P. Hunt R. H. (2016). The toronto consensus for the treatment of *Helicobacter pylori* infection in adults. *Gastroenterology* 151 51–69.e14. 27102658 10.1053/j.gastro.2016.04.006

[B14] Fiorini G. Zullo A. Saracino I. M. Pavoni M. Vaira D. (2018). Antibiotic resistance pattern of *Helicobacter pylori* strains isolated in Italy during 2010-2016. *Scand. J. Gastroenterol.* 53 661–664. 29688095 10.1080/00365521.2018.1464596

[B15] Ford A. C. Forman D. Hunt R. H. Yuan Y. Moayyedi P. (2014). *Helicobacter pylori* eradication therapy to prevent gastric cancer in healthy asymptomatic infected individuals: systematic review and meta-analysis of randomised controlled trials. *BMJ* 348:g3174. 10.1136/bmj.g3174 24846275 PMC4027797

[B16] Gerrits M. M. van Vliet A. H. Kuipers E. J. Kusters J. G. (2006). *Helicobacter pylori* and antimicrobial resistance: molecular mechanisms and clinical implications. *Lancet Infect Dis.* 6 699–709. 10.1016/S1473-3099(06)70627-2 17067919

[B17] Giorgio F. Ierardi E. Sorrentino C. Principi M. Barone M. Losurdo G. (2016). *Helicobacter pylori* DNA isolation in the stool: an essential pre-requisite for bacterial noninvasive molecular analysis. *Scand. J. Gastroenterol.* 51 1429–1432. 10.1080/00365521.2016.1216592 27687850

[B18] Kjøller M. Fischer A. Justesen T. (1991). Transport conditions and number of biopsies necessary for culture of *Helicobacter pylori*. *Eur. J. Clin. Microbiol. Infect. Dis.* 10 166–167. 10.1007/BF01964450 2060517

[B19] Liou J. M. Lin J. T. Chang C. Y. Chen M. J. Cheng T. Y. Lee Y. C. (2010). Levofloxacin-based and clarithromycin-based triple therapies as first-line and second-line treatments for *Helicobacter pylori* infection: a randomised comparative trial with crossover design. *Gut* 59 572–578. 10.1136/gut.2009.198309 20427390

[B20] Liu W. Z. Xie Y. Lu H. Cheng H. Zeng Z. R. Zhou L. Y. (2018). Fifth chinese national consensus report on the management of *Helicobacter pylori* infection. *Helicobacter* 23:e12475. 10.1111/hel.12475 29512258

[B21] López-Góngora S. Puig I. Calvet X. Villoria A. Baylina M. Muñoz N. (2015). Systematic review and meta-analysis: susceptibility-guided versus empirical antibiotic treatment for *Helicobacter pylori* infection. *J. Antimicrob Chemother.* 70 2447–2455. 10.1093/jac/dkv155 26078393

[B22] Malfertheiner P. Megraud F. O’Morain C. A. Atherton J. Axon A. T. Bazzoli F. (2012). Management of *Helicobacter pylori* infection–the Maastricht IV/florence consensus report. *Gut* 61 646–664. 10.1136/gutjnl-2012-302084 22491499

[B23] Malfertheiner P. Megraud F. O’Morain C. A. Gisbert J. P. Kuipers E. J. Axon A. T. (2017). Management of *Helicobacter pylori* infection-the maastricht V/Florence consensus report. *Gut* 66 6–30. 10.1136/gutjnl-2016-312288 27707777

[B24] McColl K. E. (2010). Clinical practice. *Helicobacter pylori* infection. *N. Engl. J. Med.* 362 1597–1604. 20427808 10.1056/NEJMcp1001110

[B25] Mégraud F. Lehours P. (2007). *Helicobacter pylori* detection and antimicrobial susceptibility testing. *Clin. Microbiol. Rev.* 20 280–322. 17428887 10.1128/CMR.00033-06 PMC1865594

[B26] Midolo P. D. Bell J. M. Lambert J. R. Turnidge J. D. Grayson M. L. (1997). Antimicrobial resistance testing of *Helicobacter pylori*: a comparison of Etest and disk diffusion methods. *Pathology* 29 411–414. 10.1080/00313029700169415 9423225

[B27] Miura S. Hokari R. (2012). Seeking an optimal eradication therapy for *Helicobacter pylori* infection. *J. Gastroenterol. Hepatol.* 27 7–9. 10.1111/j.1440-1746.2011.06953.x 22188025

[B28] Nezami B. G. Jani M. Alouani D. Rhoads D. D. Sadri N. (2019). *Helicobacter pylori* mutations detected by next-generation sequencing in formalin-fixed, paraffin-embedded gastric biopsy specimens are associated with treatment failure. *J. Clin. Microbiol.* 57:e01834-18. 10.1128/JCM.01834-18 31068413 PMC6595463

[B29] O’Connor A. O’Morain C. A. Ford A. C. (2017). Population screening and treatment of *Helicobacter pylori* infection. *Nat. Rev. Gastroenterol. Hepatol.* 14 230–240. 28053340 10.1038/nrgastro.2016.195

[B30] Palmitessa V. Monno R. Panarese A. Cuppone R. Burattini O. Marangi S. (2020). Evaluation of antibiotic resistance of *Helicobacter pylori* strains isolated in bari, southern italy, in 2017-2018 by phenotypic and genotyping methods. *Microb Drug Resist.* 26 909–917. 10.1089/mdr.2019.0262 32101078

[B31] Thung I. Aramin H. Vavinskaya V. Gupta S. Park J. Y. Crowe S. E. (2016). Review article: the global emergence of *Helicobacter pylori* antibiotic resistance. *Aliment Pharmacol. Ther.* 43 514–533. 10.1111/apt.13497 26694080 PMC5064663

[B32] Wüppenhorst N. Draeger S. Stüger H. P. Hobmaier B. Vorreiter J. Kist M. (2014). Prospective multicentre study on antimicrobial resistance of *Helicobacter pylori* in Germany. *J. Antimicrob Chemother.* 69 3127–3133. 10.1093/jac/dku243 24997315

[B33] Yahaghi E. Khamesipour F. Mashayekhi F. Safarpoor Dehkordi F. Sakhaei M. H. Masoudimanesh M. (2014). *Helicobacter pylori* in vegetables and salads: genotyping and antimicrobial resistance properties. *Biomed. Res. Int.* 2014:757941. 10.1155/2014/757941 25184146 PMC4145543

