# Supplementary material for: Correlation Analysis Among Genotype Resistance, Phenotype Resistance, and Eradication Effect After Resistance-Guided Quadruple Therapies in Refractory Helicobacter pylori Infections
Source: Front Microbiol. 2022 Mar 7;13:861626. doi: 10.3389/fmicb.2022.861626 (PMC8940283; doi:10.3389/fmicb.2022.861626)
Supplement: Supplementary file 1 [file Table_1.DOCX]

## Supplementary Table

Supplementary Table 1. Summary of main antibiotic resistance‐conferring mutations in *H. pylori*

| **Antibiotics** | **Genes** | **Major antibiotic resistance mutations** | **References** |
| --- | --- | --- | --- |
| Clarithromycin | 23S rRNA | A2143G、A2142G、A2142C、T2182C、A2144G、A2116G、A2144T、A2115G、G2111A、C2147G、G1939A、T1942C | ^34-36^ |
| Amoxicillin | PBP-1A | Substitution mutations (T556S, N562Y, T593A, S414R, A369T, V374L, L423F); insertion mutations (464+E); nonsense mutations (Y637*) | ^37-39^ |
| Levofloxacin | gyrA | D91 (G, N, A, Y), N87K, A88V, S83A, N87A | ^40^ |
| Metronidazole | rdxA | ﻿Missense mutations( ﻿R16, H17, S18, C19, K20, R41, L42, S43, Y47, Q50, V55, M56, N73, I142, A143, G145, G149, C159, G162, G163, V192, K198, K200, K202, L209); frameshift or nonsense mutations ﻿(all TfsTer, and Ter); ﻿large sequence deletions (K2_M21del, R131_K166del, K168_V172del, L137_I142del, N178_L185del; G189_R200del, S92_Q146del), large sequence insertions, missense mutations observed in clinical isolates (e.g. A22S, E27Q/V, T31E, D59N, R90K, H97T/Y, P106S, S108A, A118S/T, R131K, and G189C) | ^41-43^ |
| Tetracycline | 16S rRNA | AGA (926-928) →TTC | ^44^ |
| Furazolidone | oorD | A041G, A122G, C349A(G), A78G, A112G, A335G, C156T, C165T |  |
|  | porD | G353A, A356G, C357T, C347T, C347G, C346A |  |

Supplementary Table 2. The frequency of side effects in eradication success and failure groups

| **Side effects** | **Success group (n =31),** n (%) | **Failure group (n =4),** n (%) | ***P* value**^*^ |
| --- | --- | --- | --- |
| Nausea | 11(35.5) | 1(25.0) | 0.678 |
| Diarrhea | 6(51.5) | 2(50.0) | 0.170 |
| Metallic taste | 2(6.5) | 1(25.0) | 0.212 |
| Epigastric discomfort | 2(6.5) | 0(0.0) | 0.601 |
| Melena | 5(16.1) | 2(50.0) | 0.111 |
| Urticaria | 1(3.2) | 1(3.2) | 0.077 |

^*^All *P* values were calculated by Fisher's exact test.

Supplementary Table 3. The correlation of antibiotic regimens with adverse effects

| **Regimen** | Group with side effects (n=15), n (%) | Group without side effects (n=20), n (%) | ***P* value**^*^ |
| --- | --- | --- | --- |
| **TET+FZD** | 4 | 0 | 0.026 |
| **TET+MTZ** | 2 | 2 | 0.581 |
| **AMX+FZD** | 6 | 10 | 0.557 |
| **AMX+LVX** | 7 | 5 | 0.181 |

AMX, amoxicillin; LVX, levofloxacin; MTZ, metronidazole; TET, tetracycline; FZD, furazolidone.

^*^All *P* values were were calculated by Student's t test or Fisher's exact test.
